# Investing in the Academic Writing: Training Future Reviewers and Sustaining Efficient and Quality Peer Review

**DOI:** 10.7759/cureus.30341

**Published:** 2022-10-16

**Authors:** B.M. Munasinghe, Champa Chapman, Chinthaka Hewavitharane, Gayathri Hewawasam, T.G. Dissanayakege

**Affiliations:** 1 Anesthesiology and Critical Care, Queen Elizabeth The Queen Mother Hospital, Margate, GBR

**Keywords:** peer review, peer reviewer, reviewer residency, co-reviewing, open review, trainee reviewer, junior reviewer

## Abstract

Peer reviewers are considered gatekeepers in academic writing who play a pivotal and essential role during the publication process. Excellent manuscripts invariably need excellent reviewers. Producing peer reviewers with such caliber is time-consuming albeit necessary for the progress and continuity of academia. Despite the popular belief that an experienced author invariably makes a good reviewer, the reality is far-fetched. This suggests the need for peer reviewer training, which should be effective, logistically affordable, and demonstrate long-lasting positive impacts. Open review, co-review, and several reviewer training programs are already in place for this purpose with varying efficiencies. This narrative review discusses the current modalities available to a junior reviewer to improve his/her review skills and proposes a reviewer residency concept that could be adopted as a part of peer reviewer training.

## Introduction and background

The delay in peer review is universally identified as a leading factor of the author and editorial unease. To address this, it has become a requisite to train and mentor junior researchers by the journal itself with a system to recruit willing candidates and guide them until independent reviewing [1-3]. Co-reviewing is a concept of combined conduct of review by a junior reviewer and a senior reviewer [4]. Open review could be similarly beneficial for junior reviewers. Different levels of "reviewer residency" commencing from novice reviewers could also be formulated. A dedicated senior reviewer or an editor can act as the supervisor. Progressively complex manuscripts could be allocated to new reviewers at different levels of residency. Statistical prowess could be instilled by structured teaching, applications of which could be assessed during reviews by new reviewers. As an incentive, both the supervisor and the trainee reviewer could be offered waivers during self-submissions whenever applicable. To motivate aspiring reviewers, annual reviewer acknowledgments could be influential. Reviews of trainee reviewers that are of acceptable quality could be sent to authors. Each journal could adapt this system to have a continuous stream of reviewers of the highest standard eventually.

## Review

The modern-day peer review still lacks perfection, and its integrity and efficiency are questioned [5]. While this cast of doubt is multifaceted, delayed and/or biased peer reviews are contributory [6]. Peer reviewers often have additional administrative, teaching, and research commitments that might take precedence over altruistic review. Such busy schedules contribute to delayed reviews. In addition, the number of manuscripts required to be reviewed annually by reviewers who are authors themselves is significant [7]. These suggest the incorporation of junior academics for the peer review process, to train, guide, and supervise them, which will create a pool of competent reviewers. This cycle would ensure the availability and adequacy of peer reviewers for the future when the number of manuscripts to be reviewed will invariably increase.

This review discusses the currently available pathways for junior reviewers to achieve proficiency in peer review.

Open review

Currently, some of the leading biomedical journals such as the BMC, BMJ, and Nature Communications adopt an open review system during the review process [8]. Open peer review differs from the traditional method in several aspects. The reviewer’s identity is known to the readers and the author. Importantly the reviewer reports are published alongside the accepted manuscript [9]. It is pointed out that by open peer review, such valuable "discussions among authors and peer reviewers do not go wasted" [9], which is beneficial for potential future reviewers as reviewer reports themselves can be educational not only by factual content on the reviewed subject (as the reviewers are experts in the particular field) but also by the analytical aspects employed by the reviewers.

Co-review

Co-review denotes when a non-invited reviewer contributes with ideas or by writing during a review of a manuscript. Usually, co-reviewers are postgraduate students supervised by a senior researcher. It is found that a significant proportion of novice researchers act as co-reviewers alongside their senior researchers [4]. The same study has found that the majority of the co-reviewers found it useful in improving their competency. Co-review is being given more weight at present and is considered by some journals to be a permanent part of their peer-review process in the future [10]. These journals invite their reviewers to collaborate with junior researchers of their preference to contribute to the review process. Apart from the benefits of direct involvement and supervision, the junior reviewers get recognized for their reviews. For instance, IOPscience journals allow the principal reviewers to add co-reviewers to their Publons profiles so they can claim the review. Journals such as the International Journal of Medical students conduct an internal peer review carried out by a published medical student as a student editor supervised by an associate editor. External peer review is opted if the manuscripts are deemed suitable after this phase. Some studies have shown that such pairing of a junior reviewer with a senior reviewer improves their reviewer skills [11].

Reviewer training through editorial assessments, workshops, and learning packages

In view of improving the quality of peer review, the following had been employed by journals, and their efficacy was subsequently assessed: (1) providing feedback and quality assessment by the editor [12-14]; (2) structured training programs [15]; (3) self-instructional review packages [16]; and (4) providing online learning modules and subsequent co-review with a senior reviewer [17].

Out of these, the reviewers had positive remarks on editor feedback on improving their reviews [13,14]. They suggested the need for more detailed editorial feedback on their reviews. While there were modest improvements in identifying errors in manuscripts following the use of self-instructional packages, all measures failed to demonstrate any persistent improvements. Additionally, conducting workshops poses logistic problems with reduced access to a wider circle of junior reviewers [18]. The Annals of Emergency Medicine conducted a randomized control study to assess whether mentoring reviewers had any effect on the overall improvement of the quality of the review [17]. In line with the previous study findings, they concluded there were no positive impacts on the former. They observed that in the current academia, editorial feedback for reviewers is minimal and that it requires long-term follow-up and mentoring of junior reviewers to have a demonstrable positive effect, which might be impractical for most journals.

Access to other peer reviewer assessments

Some journals such as the Cureus Journal of Medical Science accommodate the visibility of reviews of other reviewers allocated to a manuscript. This allows a junior reviewer to improve and incorporate analytical aspects utilized by senior reviewers during the review process.

Reviewer residency programs

We propose a reviewer residency program to be adopted by each journal to recruit, train, and sustain a pool of peer reviewers. This consists of several stages.

Identifying Potential Reviewers Who State Willingness to Undergo Reviewer Residency

Every journal provides links to authors who are willing to become reviewers. During this initial stage, the chance to follow the residency can be offered. This should not be based on expertise and experience but on the voluntary desire of the author.

Identifying a Senior Reviewer/Editor as the Mentor

Depending on the commitments of the senior reviewers/editors, several new reviewers can be allocated for their supervision, provided they are willing. Otherwise, each could be given the responsibility of supervising a single new reviewer.

Reviewer Residency 

**Figure 1:**
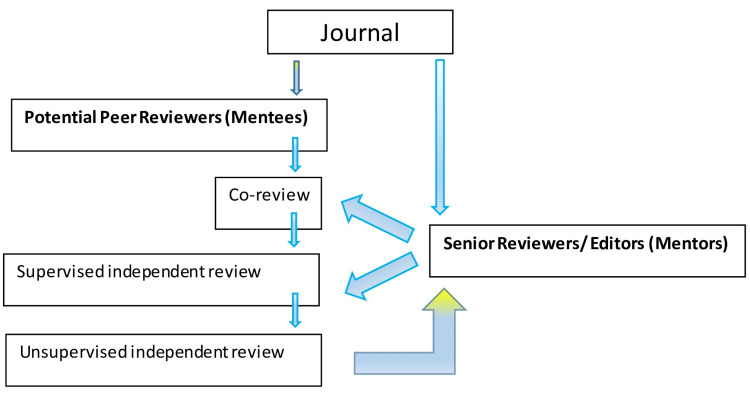
Schematic representation of a "Reviewer Residency" program

This basically suggests stepwise training, simultaneous assessment, and ultimately allowing independent reviewing by the new reviewer. This is illustrated in Figure 1.

Initial co-review: The junior reviewer is given an uncomplicated manuscript (simple research protocols, case reports, case series, etc.) to review with a standard format to be completed during the review. The format should assess the basic components required in a manuscript. Such formats are available in most of the journals at present, which will make it easier to implement the proposed method. Following completion of the review, the senior reviewer provides his/her review on the same manuscript and discusses the aspects to improve on the new reviewer report. Provided the co-review is of acceptable quality, these reviewer comments could be sent to the author and junior reviewer informed regarding re-review if required and the cycle continues. Gradually complex manuscripts (with more complex research protocols and study designs and data analysis and review articles etc.) could be assigned. In case the need for additional expertise is expressed by the new reviewer (statistical knowledge for example), they could be directed to suitable personnel (such as statisticians) who contribute to the journal and are provided with the necessary software.

Supervised independent review: The number of manuscripts to be co-reviewed should be jointly decided by the senior reviewer and the new reviewer. When both parties are satisfied, the new reviewer can be allocated to review independently supervised by the mentor. By this stage, the reviewer's comments are revised only if required and submitted to the author. The junior reviewer should be provided with comments from other external peer reviewers at both these stages, which will contribute to improving his/her reviewer skills.

Unsupervised, independent review: By this stage, the junior reviewer has mastered the basic and advanced skills and knowledge to conduct a quality peer review.

Communication between the senior reviewer and the mentees is possible nowadays with online communication such as email, mobile phone applications, and other online real-time communication applications. This will minimize delays in communication and feedback between the two groups. Similarly, to motivate and retain all categories of reviewers irrespective of their seniority, the reviewer names can be included in the published manuscript, which may include the co-reviewers as well. Provision of incentives in the form of waivers for the reviewers during the submission of their own manuscripts and reviewer/co-reviewer acknowledgments annually are all already practiced methods in the majority of the journals.

We agree with the authors who have previously suggested that such reviewer training programs might be impractical for journals. However, it is sensible to think that persistent editorial/senior reviewer mentorship will have a sustained positive effect on the reviewer output, provided that both the editor/senior reviewer and the new reviewer are responsible and conscious of the progress of the training. On the other hand, despite being a difficult task to implement such programs journal-wise, with time there will be a competent pool of reviewers and subsequent editors, which will allow the system to self-sustain. The provision of certificates toward the completion of reviewer residency which is accepted in academia would make such programs well-accepted and sought out.

## Conclusions

Peer reviewer training is imperative in modern times with more manuscripts presented and the competition to publish grows. The current models of peer reviewer training are evolving, and their impact will be analyzed in the future. Peer reviewer residency is a concept that incorporates all these practiced models in view of producing quality reviewers and the sustenance of the review process. Despite already cited drawbacks, such as the practical difficulties of such programs, modern technology has provided alternatives, such as freely accessible communication applications and free software, which would provide solutions to overcome these and continue such programs. It is time to consider adopting these in all journals. As the great Walt Disney remarked, "We keep moving forward, opening new doors, and doing new things, because we're curious and curiosity keeps leading us down new paths," the academia moves on, and so should the peer review.

## References

[1] Alam S, Patel J (2015). Peer review: tips from field experts for junior reviewers. BMC Med.

[2] Garisto D (2019). Nature Index. Diversifying peer review by adding junior scientists. Nat Index.

[3] Jamali HR, Nicholas D, Watkinson A (2020). Early career researchers and their authorship and peer review beliefs and practices: an international study. Learn Publ.

[4] McDowell GS, Knutsen JD, Graham JM, Oelker SK, Lijek RS (2019). Co-reviewing and ghostwriting by early-career researchers in the peer review of manuscripts. Elife.

[5] Tennant JP, Ross-Hellauer T (2020). The limitations to our understanding of peer review. Res Integr Peer Rev.

[6] Tosi H (2009). It's about time!!!!: What to do about long delays in the review process. J Manag Inq.

[7] Fernandez-Llimos F, Salgado TM, Tonin FS (2020). How many manuscripts should I peer review per year?. Pharm Pract (Granada).

[8] Ross-Hellauer T, Görögh E (2019). Guidelines for open peer review implementation. Res Integr Peer Rev.

[9] Ross-Hellauer T (2017). What is open peer review? A systematic review. F1000Res.

[10] East AE, Attal M, Hoitink AJ, Sergienko OV (2022). Thank you to our 2021 reviewers, and a new co‐reviewing protocol. J Geophys Res Earth Surf.

[11] Min HT (2005). Training students to become successful peer reviewers. System.

[12] Callaham ML, Knopp RK, Gallagher EJ (2002). Effect of written feedback by editors on quality of reviews: two randomized trials. JAMA.

[13] Freda MC, Kearney MH, Baggs JG, Broome ME, Dougherty M (2009). Peer reviewer training and editor support: results from an international survey of nursing peer reviewers. J Prof Nurs.

[14] Tercier Tercier, J. J., & Callaham, M. L. (2007). UCSF: Department of Emergency Medicine. A normative model of peer review: qualitative assessment of manuscript reviewers’ attitudes towards peer review. https://escholarship.org/uc/item/4p90p67x.

[15] Callaham ML, Wears RL, Waeckerle JF (1998). Effect of attendance at a training session on peer reviewer quality and performance. Ann Emerg Med.

[16] Schroter S, Black N, Evans S, Carpenter J, Godlee F, Smith R (2004). Effects of training on quality of peer review: randomised controlled trial. BMJ.

[17] Houry D, Green S, Callaham M (2012). Does mentoring new peer reviewers improve review quality? A randomized trial. BMC Med Educ.

[18] Callaham ML, Schriger DL (2002). Effect of structured workshop training on subsequent performance of journal peer reviewers. Ann Emerg Med.

